# Estimating heterogeneous treatment effect by balancing heterogeneity and fitness

**DOI:** 10.1186/s12859-018-2521-7

**Published:** 2018-12-31

**Authors:** Weijia Zhang, Thuc Duy Le, Lin Liu, Jiuyong Li

**Affiliations:** 1 School of Information Technology and Mathematical Sciences, University of South Australia, Adelaide, Australia; 2 Centre for Cancer Biology, University of South Australia, Adelaide, Australia

**Keywords:** Heterogeneous treatment effect, Breast cancer, Radiotherapy

## Abstract

**Background:**

Estimating heterogeneous treatment effect is a fundamental problem in biological and medical applications. Recently, several recursive partitioning methods have been proposed to identify the subgroups that respond differently towards a treatment, and they rely on a fitness criterion to minimize the error between the estimated treatment effects and the unobservable ground truths.

**Results:**

In this paper, we propose that a heterogeneity criterion, which maximizes the differences of treatment effects among the subgroups, also needs to be considered. Moreover, we show that better performances can be achieved when the fitness and the heterogeneous criteria are considered simultaneously. Selecting the optimal splitting points then becomes a multi-objective problem; however, a solution that achieves optimal in both aspects are often not available. To solve this problem, we propose a multi-objective splitting procedure to balance both criteria. The proposed procedure is computationally efficient and fits naturally into the existing recursive partitioning framework. Experimental results show that the proposed multi-objective approach performs consistently better than existing ones.

**Conclusion:**

Heterogeneity should be considered with fitness in heterogeneous treatment effect estimation, and the proposed multi-objective splitting procedure achieves the best performance by balancing both criteria.

## Background

Treatment effect estimation is a fundamental problem in scientific research. Biologists use it to study the regulatory relationships between numerous genes [1], and medical researchers rely on it to determine whether a treatment is effective for the patients [2].

Traditionally, the treatment effect is estimated as an average value for the entire population. However, understanding the heterogeneity of treatment effects are important for many applications. For example, although radiotherapy is an effective treatment for cancer patients in general, some of the patients do not benefit from it because of their different gene expression patterns [3].

It is desirable to apply principled data mining methods to inference the heterogeneity in the treatment effects [4]. Tree-based recursive partitioning methods [5], originally proposed for regression and classification, are perfect candidates for modeling treatment effect heterogeneity. Unlike methods which have strong predictive power but are difficult to interpret, tree-based methods often excel on both frontiers. Their output, tree models, can be easily interpreted by human experts, which is of an important consideration in both biological and medical applications.

A fundamental impediment must be cleared before recursive partitioning methods can be applied to estimate heterogeneous treatment effects. In regression and classification, the target variables are available in the training data. Unfortunately, such information is almost never available in treatment effect estimation because a sample can either be treated or not treated. In other words, only one of the two potential outcomes is observable but both outcomes are needed for the estimation [6].

Recently, a number of recursive partitioning methods have been proposed to solve the problem by utilizing the fitness criterion [7–9]. Specifically, these methods employ a surrogate loss function to minimize the error between the estimated treatment effects and the unobservable ground truth treatment effects.

**Fig. 1:**
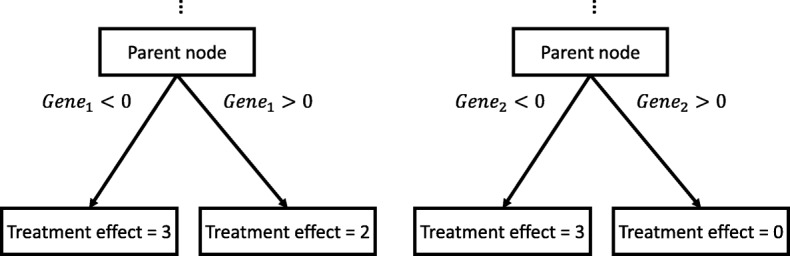
Illustration of two models for estimating heterogeneous treatment effect. The first model preferred fitness while the second one prioritized heterogeneity

Understanding the heterogeneity of treatment effect has important real-world implications. For example, consider two models describing the radiotherapy treatment effect for breast cancer patients (Fig. 1). The first model divides the patients into two sub-populations according to the expression level of *gene*_1_, and the second model places the split at *gene*_2_. If the errors of both models are within an acceptable level, the second model should be preferred because it shed more lights on how the treatment effects vary among different subpopulations of the patients.

**Fig. 2:**
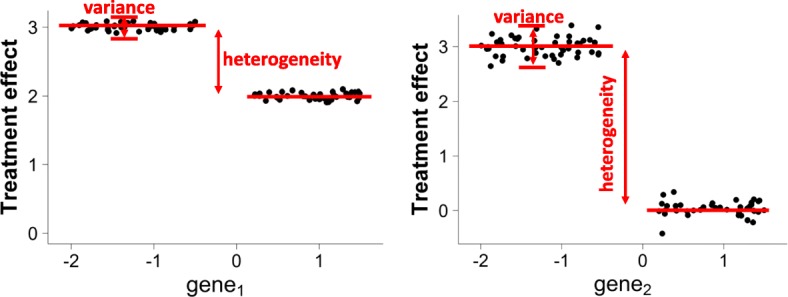
Estimated treatment effects for models in Fig. 1. When the sub-populations are split at *gene*_2_, the estimated error is slightly larger than splitting at *gene*_1_. This explains why existing methods prefer Model 1. However, the heterogeneity of treatment effects is ignored in this criterion

Heterogeneity should be considered explicitly during the recursive partitioning process. As illustrated in Fig. 2, although the first model has slightly lower estimated error than the second one, it provides less insight on treatment effect heterogeneity than the second model. In this example, existing methods will prefer the first model and fail to revealing the heterogeneity because the heterogeneity is not considered.

Without explicitly maximizing heterogeneity, a criterion may favor splits resulting in homogeneous nodes. Producing homogeneous nodes is problematic for applications where the number of samples are limited, which is almost ubiquitous in bioinformatics problems. A split with homogeneous nodes will halve the sample size without providing insight to the treatment effect heterogeneity, and the reliability of treatment effect estimation for the subsequent splits will decrease. A closely related method, namely the Causal Tree (CT) [7], does not explicitly maximize the heterogeneity and often produce split leading to homogeneous nodes.

Moreover, heterogeneity and fitness need to be considered simultaneously during the splitting procedure. If the splitting is based solely on the heterogeneity criterion, the algorithm will be prone to favor models with spuriously high treatment effect differences but unacceptably estimation errors. Finding the optimal splits should be considered as a multiple-objective problem: the first objective is to maximize the fitness (minimize the estimated errors of treatment effect) and the second objective is to maximize the heterogeneity.

In this paper, we first propose the Maximizing Heterogeneity (MH) splitting criterion for heterogeneous treatment effect estimation under the recursive partitioning framework. Then we propose the multi-objective (MO) splitting procedure to consider both the heterogeneities and the fitnesses when building a recursive partitioning model. When solutions which maximize heterogeneity and fitness simultaneously are not achievable, MO aims to strike a balance between both criteria by allowing a certain degree of slack into their dominance relationships.

We compare the proposed methods with existing methods using both simulated and real-world datasets. Experiment results demonstrate that while MH performs better than existing ones in many cases, it is prone to error when the differences in treatment effects become small among subgroups. When fitness and heterogeneity are balanced, MO performs consistently better than all compared methods.

## Methods

### Preliminaries

In this section, we introduce necessary definitions and results for heterogeneous treatment effect estimation.

Let *W*_*i*_∈{0,1} denote the treatment assignment, *Y*_*i*_ denote the observed outcome, and **x**_*i*_={*x*_*i*1_,…,*x*_*ip*_} denote the pre-treatment covariates. The data consists of i.i.d. samples (*Y*_*i*_,*W*_*i*_,**x**_*i*_), for *i*=1,…,*N*. For the sake of simplicity, the subscript *i* will be omitted when the context is clear.

Let *Y*^(*W*)^ denote the potential outcome if an individual has received the treatment *W*, then the observed outcome *Y* can be described as *Y*=*WY*^(1)^+(1−*W*)*Y*^(0)^. Although each sample is associated with two potential outcomes *Y*^(1)^ and *Y*^(0)^, only one of them can be realized as the observed outcome *Y*.

The average treatment effect (ATE) is defined as the expected outcome if the entire population were treated minus the outcome if they were not treated [6]:

1 $$  \tau=\mathbb{E}\left[Y^{(1)} - Y^{(0)}\right].  $$

Since only one of the two potential outcomes can be observed, Equation 1 is *counterfactual* and cannot be estimated straightforwardly. When the treatment assignment is completely random, i.e., , the average treatment effect can be estimated as $\tau =\mathbb {E}(Y|W=1)-\mathbb {E}(Y|W=0)$.

However, the treatment assignment is often not randomized. In such cases, the unconfoundedness assumption [6] is needed in order to estimate treatment effect in these circumstances:

#### **Assumption 1**



With the assumption, an unbiased ATE estimation can be achieved with the help of propensity score [10]. The propensity score is defined as *e*(**x**)=*Pr*(*W*=1|**x**), the probability of treatment assignment conditioning on the covariates.

The propensity score can then be estimated with a variety of methods. Some popular choices include logistic regression, random forests, and boosting [11].

When treatment effects are heterogeneous across the population, estimating the conditional average treatment effect (CATE) [6] in various subpopulations defined by the possible values of the covariates **x** often provides more insight than estimating the ATE on the entire population. Specifically, CATE is defined as:

$$\tau(\mathbf{x})=\mathbb{E}[Y(1)-Y(0)|\mathbf{x}]. $$

Recursive partitioning provides an ideal way for estimating CATE. Starting from the root node containing the entire population, a tree model is constructed by recursively splitting the node into two disjoint child nodes. By the end of the procedure, the subpopulations with heterogeneous treatment effects are naturally presented in the leaves of the model. For each leaf node, *τ*(**x**) can be estimated by calculating the ATE using only the samples within the node as follows:

2 $$  \hat\tau(\mathbf{x})=\frac{\sum\limits_{\mathbf{x}_{i} \in \mathcal{N}}^{n}\frac{W_{i}\cdot Y_{i}}{e\left(\mathbf{x}_{i}\right)}}{\sum\limits_{\mathbf{x}_{i} \in \mathcal{N}}^{n}\frac{W_{i}}{e\left(\mathbf{x}_{i}\right)}} -\frac{\sum\limits_{\mathbf{x}_{i} \in \mathcal{N}}^{n}\frac{\left(1-W_{i}\right)\cdot Y_{i}}{\left(1-e\left(\mathbf{x}_{i}\right)\right)}}{\sum\limits_{\mathbf{x}_{i} \in \mathcal{N}}^{n}\frac{1- W_{i}}{\left(1-e\left(\mathbf{x}_{i}\right)\right)}},  $$

where the treatment propensity *e*(**x**_*i*_) is either known from experimental design or estimated from observational data.

The core component of a recursive partitioning model is the splitting criterion. At each split, the splitting criterion relies on a scoring function to evaluate the qualities of all potential splitting points. The recursive partitioning model then makes the split at the splitting point with the highest score.

The fitness criterion, one of the most widely adopted splitting criteria, aims to maximize the fitness of the model by minimizing the mean squared error(MSE). However, since the true treatment effects are not observable, the MSE cannot be estimated straightforwardly. In [7, 9], the authors observed that under Assumption 1, it can be obtained that

3 $$ \mathbb{E} \left[\tau_{i} | i\in \mathcal{N}\right] = \mathbb{E}[ \hat{\tau} (\mathbf{x}|\mathbf{x} \in \mathcal{N})]   $$

Relying on Eq. 3, [7] has proposed to utilize an alternative scoring function to estimate the error as:

4 $$ \mathcal{C}^{fit} := n_{L} \cdot \hat{\tau}_{L}^{2} + n_{R}\cdot \hat{\tau}_{R}^{2},   $$

where *τ*_*L*_ and *τ*_*R*_ are the estimated treatment effects, *n*_*L*_ and *n*_*R*_ are the numbers of samples in the left and right child node.

### The proposed multi-objective splitting criterion

A problem of the fitness criterion $\mathcal {C}^{fit}$ is that the expectation equation in 3 is only valid when the sample size is sufficiently large. Unfortunately, in recursive partitioning the sample size of a node becomes more and more smaller than the sample size of the original dataset *N* as the tree grows. To make things worse, this problem is amplified in biological and medical researches, where *N* is already small relative to the number of variables.

From the examples in Figs. 1 and 2, it is conceivable that explicitly considering heterogeneity is beneficial for recursive partitioning model construction.

Therefore, we propose that a heterogeneity criterion, which maximizes the differences in treatment effects of the child nodes, also needs to be considered for recursive partitioning. Specifically, the proposed heterogeneity criterion favors the split with the largest treatment effect heterogeneity in the subpopulations of the child nodes:

5 $$ \mathcal{C}^{hete} := \left(\hat{\tau}_{L} - \hat{\tau}_{R} \right)^{2},   $$

In Fig. 2, a recursive partitioning method utilizing $\mathcal {C}^{hete}$ will choose *gene*_2_ over *gene*_1_. Because when splitting at *gene*_2_, since it results in larger heterogeneity in treatment effects than splitting at *gene*_1_. In the following sections, we will refer the criterion in Eq. 5 as Maximizing Heterogeneity (MH).

As will be demonstrated in the next section, relying only on the MH criterion achieves better performances than using the fitness criterion in many cases. But still, there are circumstances where the MH criterion would achieve worse performances than the fitness criterion.

This is caused by the fact that the MH criterion does not place any consideration on the fitness of the model. In other words, the MH criterion would select a splitting point with high heterogeneity even if it also has high mean squared error.

Consider the example in Fig. 2, suppose there exists another covariate *gene*_3_ and splitting at *gene*_3_ achieves higher heterogeneity than splitting at *gene*_2_, but also has higher mean squared error, then an algorithm relying only on the MH criterion will split at *gene*_3_ despite the unacceptably high MSE.

Therefore, an ideal splitting point should achieve the highest quality in terms of both the fitness criterion $\mathcal {C}^{fit}$ and the heterogeneity criterion $\mathcal {C}^{hete}$. Unfortunately, such solutions are often not available in real-world applications.

To solve this problem, we further propose splitting criterion based on multi-objective optimization to search for the most suitable splitting point. Specifically, the multi-objective criterion does not seek splitting points with the highest heterogeneity or fitness, but prefers one with a balanced fitness and heterogeneity scores.

Let $\mathbf {s}_{i} = \left (\mathcal {C}^{fit}_{i},\mathcal {C}^{hete}_{i}\right)$ denote a fitness and heterogeneity scores pair for the *i*-th possible splitting point, and let $\mathcal {S}$ be the set containing the score corresponding to all the potential split points. The goal then becomes finding the optimal *s*_*i*_ from $\mathcal {S}$. To achieve this, a dominance relationship over the set $\mathcal {S}$ needs to be defined.

Pareto dominance is a popular choice when it comes to multi-objective optimization [12]. A score vector is said to Pareto-dominate another one if and only if all its components are not smaller than the others, and at least one of its component is larger than that of the others. Then the Pareto set is an unique set which contains all the vectors that are not Pareto-dominated by any other vectors.

Despite its popularity, the original Pareto dominance concept is not suitable for our problem for two reasons. Firstly, Pareto set often contains substantial size of elements; therefore, not only are they often prohibitive to optimize, but also creates difficulties for how to choose from. Secondly, the definition of Pareto dominance does not allow the “trade-off between” among scores.

We propose an extension of the Pareto dominance relationship to achieve our objective, the *ε*-dominance relationship [13].

#### **Definition 1**

(**ε**-dominance) Score pair $s_{i} \in \mathcal {S}$is said to *ε*-dominate $s_{j}\in \mathcal {S}$ for some **ε**=(*ε*_1_,*ε*_2_), denoted as *s*_*i*_>_*ε*_*s*_*j*_, if and only if:

$$\left(1+\epsilon_{1}\right) \mathcal{C}^{fit}_{i} \geq \mathcal{C}^{fit}_{j}, \left(1+\epsilon_{2}\right)\mathcal{C}^{hete} \geq \mathcal{C}^{hete}_{j}. $$

**Fig. 3:**
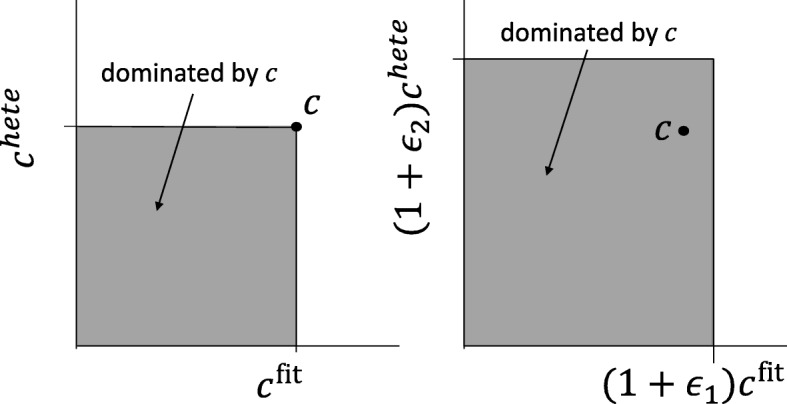
Comparison of Pareto-dominance and *ε*-dominance

The *ε*-dominance enables the capability of specifying a magnitude of difference for the different criteria (Fig. 3). Intuitively, in order for score pair *s*_*i*_ to not be *ε*-dominated by *s*_*j*_, both of *s*_*i*_’s components must be at least larger than *s*_*j*_ by a margin specified by *ε*.

With Definition 1, the *ε*-optimal set $\mathcal {S}^{\star }$ of $\mathcal {S}$ is defined as the subset $\mathcal {S}$ where all elements in *S* is *ε*-dominated by at least one element of $\mathcal {S}^{\star }$, and all elements in $\mathcal {S}^{\star }$ are in the Pareto-set of $\mathcal {S}$:

#### **Definition 2**

(*ε*-optimal set) Let $S\subseteq \mathbb {R}^{2}$ be a set of score vectors. Then the *ε*-splitting set *S*^⋆^ is defined as follows:

1. Any score *s*∈*S* is *ε*-dominated by at least one score *s*^⋆^∈*S*^⋆^, i.e.
  $$\forall s \in S: \exists s^{\star} \in S^{\star} \quad \textrm{such that} \quad s^{\star} >_{\epsilon} s, $$
2. Every score *s*^⋆^∈*S*^⋆^ are not Pareto-dominated by any score *s*∈*S*, i.e.
  $$\forall s^{\star} \in S^{\star}: \nexists s\in S \quad \textrm{such that} \quad s > s^{\star}. $$

**Fig. 4:**
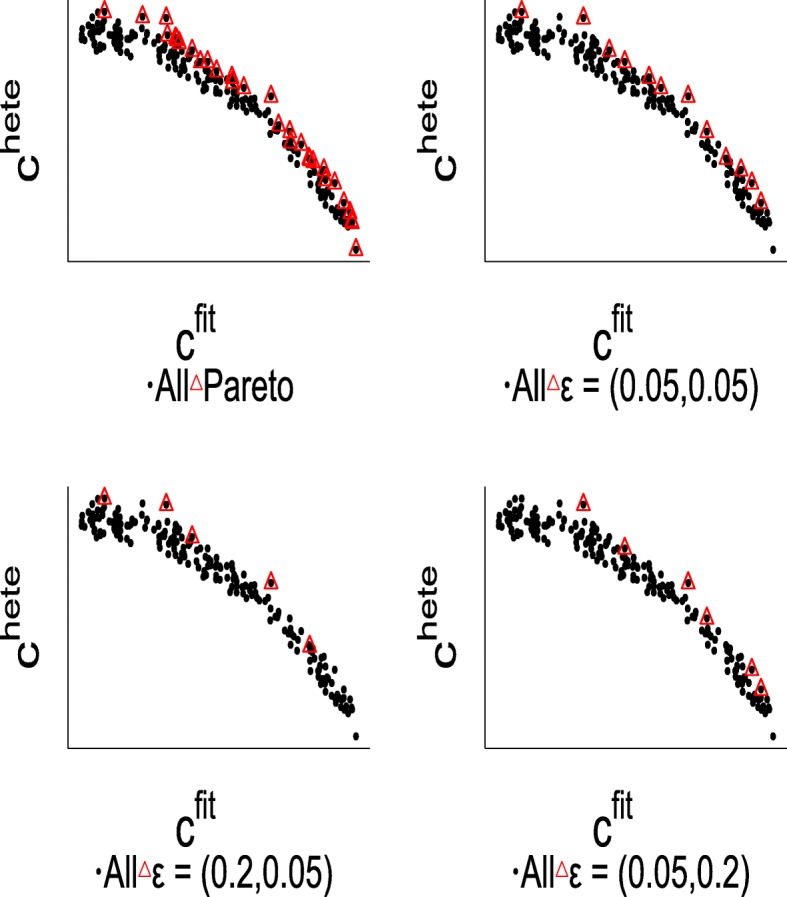
Comparison of Pareto-set and the *ε*-optimal set with various *ε* values

Comparison of Pareto-set and *ε*-optimal set are illustrated in Fig. 4, the top left panel depicts the elements in $\mathcal {S}$ and its corresponding Pareto-set, and other panels describe the *ε*-optimal set with various *ε*. Compared to the Pareto-set, *ε*-optimal set contains significantly smaller number of elements. When *ε* is sufficiently small, the *ε*-optimal set is equivalent to the Pareto-optimal set [14].

With Definitions 1 and 2, we now discuss how to maintain the *ε*-optimal set while scanning through all the potential split points without too much extra computational costs. This is achieved by dividing the two dimension search space into squares of size $\left (\left \lfloor \frac {\log \mathcal {C}^{fit}}{\log \left (1+\epsilon _{1}\right)} \right \rfloor, \left \lfloor \frac {\log \mathcal {C}^{hete}}{\log \left (1+\epsilon _{2}\right)} \right \rfloor \right)$, and only keeps one element which are not *ε*-dominated by others within the box. We present the details in Algorithm 1.

Algorithm 1 has two important properties. Firstly it is guaranteed to converge to the *ε*-optimal set. Secondly, it is guaranteed that the algorithm only needs to deal with a small number of score pairs. Formally, we summarize these properties in the following theorem.


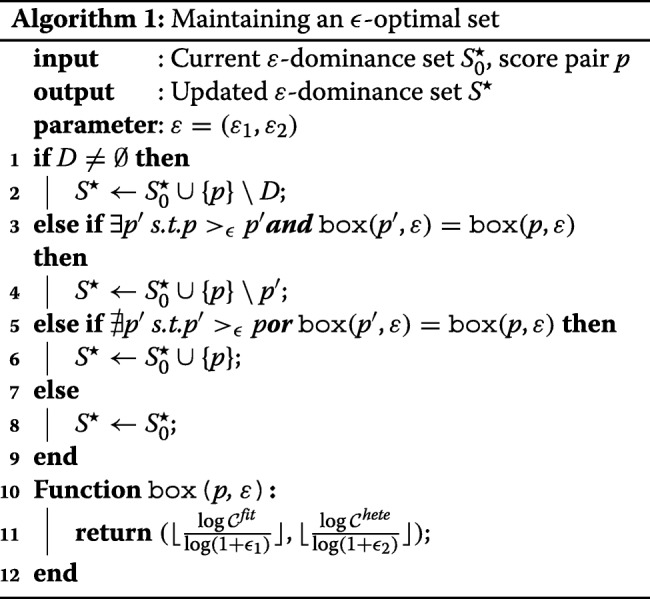


#### **Theorem**

Let *S* be the set of all score pairs for all possible splitting points. Then the output of Algorithm *1*, *S*^⋆^ is an *ε*-optimal set of *S* with bounded size:

$$\left|S^{\star}\right| = \frac{0.48}{\log\left(1+\varepsilon_{1}\right)\log \left(1+ \varepsilon_{2}\right)}. $$

#### *Proof Sketch*

On the coarse level, the search space is discretized into two-dimensional squares of size $\left (\left \lfloor \frac {\log \mathcal {C}^{fit}}{\log \left (1+\epsilon _{1}\right)} \right \rfloor, \left \lfloor \frac {\log \mathcal {C}^{hete}}{\log \left (1+\epsilon _{2}\right)} \right \rfloor \right)$, where each vector uniquely belongs to one of the squares. Applying the *ε* dominance relation on these spaces, the algorithm always maintains a set of non-dominated squares, thus guaranteeing the *ε*-optimal property. On the fine level at most one element is kept as a representative vector in each square. Within a square, the representative vector can only be replaced another one if it is *ε*-dominated, thus guaranteeing convergence. □

An important benefit of this result is that the size of set *S*^⋆^ is small and irrelevant of the total number of score pairs. For example, if *ε*_1_ and *ε*_2_ are set to be 0.2, then the upper bound of *S*^⋆^ is 75. Since Algorithm 2 needs to run at each splitting point, its time complexity is crucial to the overall running time of the MO. Fortunately, since the size of the candidate set is bounded, the time complexity of the search procedure is not affected by the number of possible split points.

Although the *ε*-optimal set is guaranteed to be of a small size, we still need to select one splitting point from its elements. According to our experiment, choosing the one with maximum $\mathcal {C}^{hete}$ achieves the best performance. Because the true treatment effect is unobservable, the cross-validation procedure also cannot be conducted straightforwardly as standard regression methods. In this work, we follow the method proposed in [7] for cross-validation.

Finally, we summarize the multi-objective tree construction procedure in Algorithm 2. The structure of splitting procedure remains similar to the CART [5] method. However, instead of only evaluate the fitness, the multi-objective criterion computes both the $\mathcal {C}^{fit}$ score and the proposed $\mathcal {C}_{hete}$ score at the same time. Then it updates the *ε*-optimal set and continues the usual splitting routine.


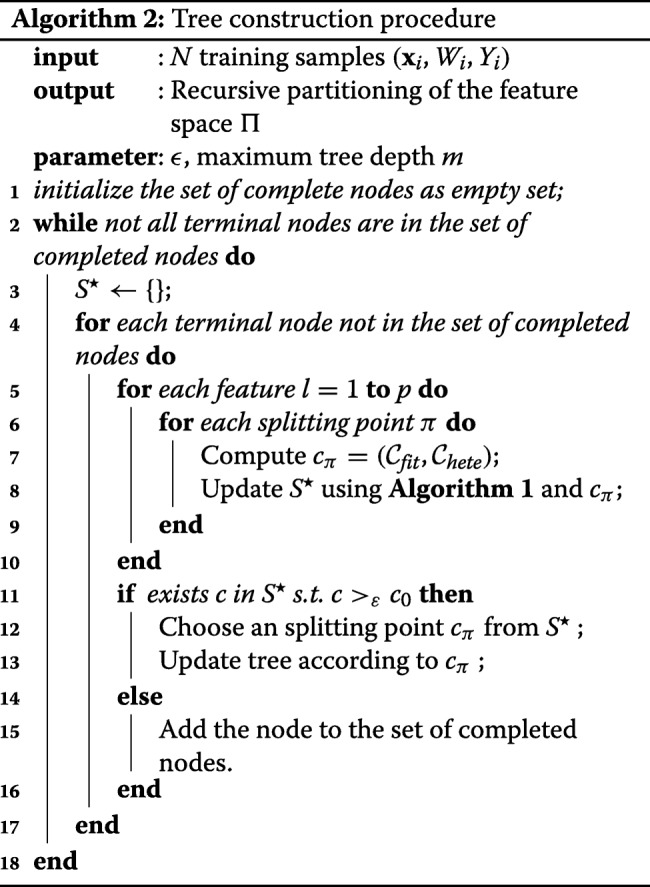


## Results

In this section we compare the performances of different splitting criteria in the recursive partitioning treatment effect estimation methods: Regression Tree (RT) [5], Transformed Outcome Tree (TOT) [6], Causal Tree (CT) [7], T-Statistic Tree (TS) [8], the proposed Maximizing Heterogeneity criterion (MH) and Multi-Objective criterion (MO).

### Synthetic data

Because the underlying treatment effects are generally inaccessible in most real-world data, we first evaluate the performance using synthetic data with the known ground truth.

We generate a group of 4 synthetic datasets to compare the performance of the proposed MH and MO criteria against existing algorithms. To ensure a fair comparison, the simulations are designed in a similar way of those used in [7, 8].

In all simulations, we satisfy Assumption 1 and the propensity score is set as *P*=0.5. The data generation mechanism is specified by the following functions:

$$\begin{array}{*{20}l} &m(x) = \frac{1}{2} \mathbb{E}\left[Y^{(0)} + Y^{(1)} | X = x\right], \\ &\tau(x) = \mathbb{E}\left[Y^{(1)} -Y^{(0)} | X = x\right].  \end{array} $$

*m*(*x*) is responsible for the mean effect which is not affected by the treatment, and *τ*(*x*) is responsible for the treatment effect. Then, the data is described as *Y*=*m*(*x*)+*α*·(2*w*−1)·*τ*(*x*)+*σ* where *α* is a parameter which controls the magnitude of the treatment effect, and *σ* is the random noise from a normal distribution.

In the first design, *α*_1_ is set to 0.5, *m*(*x*) and *τ*(*x*) include interactions among two variables:

$$m_{1}(x) = \frac{1}{2} x_{1} + x_{2},\quad \tau_{1}(x) = \frac{1}{2} x_{1}. $$

In the second design, there are 20 variables where 12 of them are noise variables which are not related to the outcome. Specifically, *α*_2_=1 and the functions are defined as:

$$\begin{array}{*{20}l} &m_{2}(x) = \frac{1}{2} \sum\limits_{k=1}^{4}x_{k} + \sum\limits_{k=5}^{8} x_{k}; \\ &\tau_{2}(x) = \sum\limits_{k=1}^{4} 1\left\{x_{k} > 0\right\}\cdot x_{k}, \end{array} $$

where 1{*x*>0} is the indicator function.

In the third design, the main and treatment functions are as similar as the second design except that the number of noise variables is increased to 50, and the value of *α*_3_ is set to 1.

$$m_{3}(x) = \frac{1}{2} \sum\limits_{k=1}^{4}x_{k} + \sum\limits_{k=5}^{8} x_{k}; \tau(x) = \sum\limits_{k=1}^{4} 1\left\{x_{k} > 0\right\}\cdot x_{k}, $$

The last design simulates non-linear treatment effect. The total number of variables is 20, and the main and treatment effect functions are defined as:

$$\begin{array}{*{20}l} &m_{4}(x) = \frac{1}{2} \sum\limits_{k=1}^{4}x_{k} + \sum\limits_{k=5}^{8} x_{k},\\ &\tau_{4}(x) = \sum\limits_{k=1}^{4} sin\left(x_{k}\right) + \sum\limits_{k=5}^{8} x_{k}^{k-3}. \end{array} $$

Two performance measurements are used to evaluate the compared methods. The first one is the root mean square error (RMSE) defined as:

$$RMSE(\hat{\tau}) = \sqrt{\frac{1}{n_{test}}\sum\limits_{i = 1}^{n_{test}}\left(\hat{\tau}\left(\mathbf{x}_{i}\right)-\tau_{i}\right)^{2}}. $$

The second criterion is the weighted root mean square error (wRMSE), where the weight is 0.1 if the estimated and the true treatment effects are of the same signs and 1 if they are of the opposite signs. Specifically,

$$wRMSE(\hat{\tau}) = \sqrt{\frac{1}{n_{test}}\sum\limits_{i=1}^{n_{test}}\omega \cdot\left(\hat{\tau}\left(\mathbf{x}\right)-\tau_{i}\right)^{2}}, $$

where *ω* = 1 if $\tau (x)\hat {\tau }(x)<0$, and *ω* = 0.1 for $\tau (x)\hat {\tau }(x) >0$.

The wRMSE measurement is particularly important in human-related studies. For example, although the cost of predicting cats as dogs is similar as the opposite in image classification tasks, the fault of predicting a potential malign tumor as benign cost significantly more than the opposite.

To ensure that different splitting criteria are the only factors that affect the performances, all methods are compared at the same number of splits instead of using cross-validation for choosing the optimal tree depth. Because the compared methods begin to over-fit the data after their depths grow too deep, we only show the results up to the depth of 15 for each method. All results reported are the average value calculated over 100 simulation runs.

**Fig. 5:**
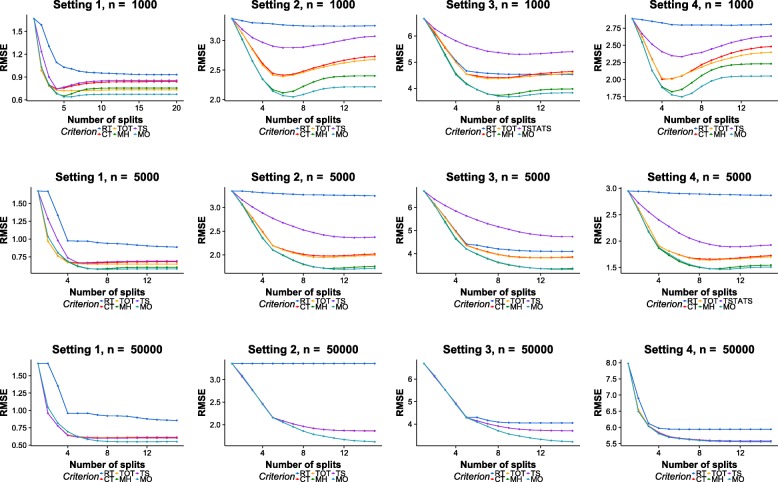
RMSE results on simulation datasets. Row: different sample sizes. Column: different experiment designs

Figure 5 shows the experimental results in terms of RMSE scores. The columns of the figure correspond to the results of different simulation designs, and rows correspond to the results of different samples sizes (*n*=1000, *n*=5000 and *n*=50000).

MH performs better than existing methods during the first few splits of the tree model. For example, in Setting 2, 3, and 4, the RMSE values of MH are lower than all existing methods at all splits. This aligns with previous observation that MH can swiftly identify the heterogeneities in treatment effects because it maximizes heterogeneity explicitly. In addition, the performance differences between MH and existing methods are larger in Setting 2 and 3 than those of Setting 1. This is possibly because that heterogeneities in treatment effects are more significant in Setting 2 and 3 than that of Setting 1 (*α*_2_,*α*_3_=0.5 and *α*_1_=1).

However, the performances of MH decrease as the tree grows deep. This is because that the heterogeneities in treatment effects become smaller as the tree grows, and MH can In Setting 1 with *n*=1000, although performs well during the first 5 splits, its RMSE values increase quickly as the tree grows deep. After the 7th split, it performs worse than TOT.

With heterogeneity and fitness both taken into consideration, MO performs consistently better than all compared methods. As can be seen from the figure, MO has the lowest RMSE values in all of the different combinations of simulation settings and sample sizes. When the sample sizes are small, the advantages of MO is the most evident. When *n*=1000, it is clear that MO is the most resistant to over-fitting.

The differences in performances become less significant as the sample sizes grow. With sufficient amount of samples, the expectation equation used in RT becomes reliable, and the chance that spurious heterogeneities mislead the MH criterion decreases. As the sample size increases, the differences between compared methods becomes smaller. At *n*=5000, TOT,TS, and CT choose exactly the same splits on all 4 settings. In the last row of the figure, the RMSE curves overlap with each other.

In most cases, the performances of existing CATE estimations methods (CT, TS, TOT) are better than the standard regression tree (RT). However, the performances of TS are worse than RT when the number of variables is large and the sample size is not sufficient, i.e., in Setting 3 when *n*=1000 and *n*=5000. This is because TS utilizes statistical tests to decide the split, which suffer from loss of power when the dimensionality grows large. In addition, the situation worsens as the sample size decreases along the tree growth.

**Fig. 6:**
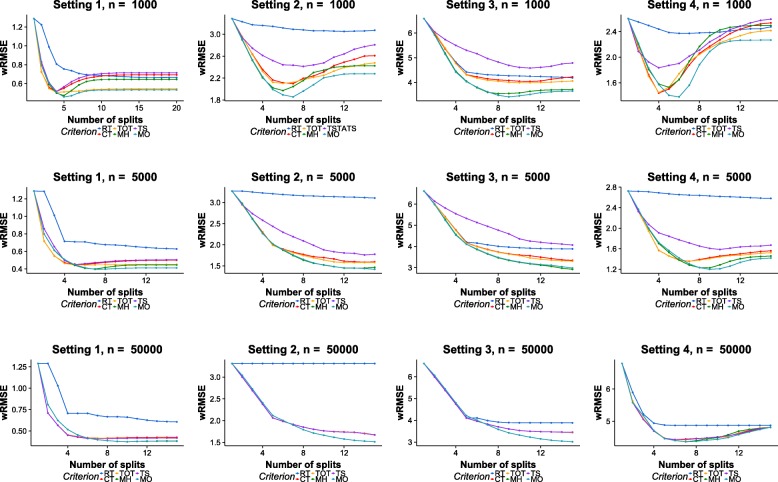
wRMSE results on simulation datasets. Row: different sample sizes. Column: different experiment designs

Figure 6 shows the wRMSE results of each method. Although the trends of performances are similar to those of RMSE, it does reveal interesting insights.

Looking at the wRMSE results with *n*=1000, the Achilles’ hell of MH is more exposed. MH performs better than existing methods during the first few splits, but its performances degenerate rapidly as the trees grow deeper. After more than 10 splits, the wRMSE measurements of MH are worse than those of TOT, CT and even RT. Again, these results confirm with the observation that MH is adept at identifying heterogeneities, but it is also prone to error caused by spuriously treatment effects.

It is also worth noticing that in some cases, existing methods have slightly lower wRMSE values than MH and MO during the first few splits. This is related to the strategy of selecting a split point from the *ε*-optimal set. As discussed in the “Methods” section, when there are multiple elements in the *ε*-optimal set, the split with the highest $\mathcal {C}^{hete}$ score is chosen from the set. However, if the splitting point with the highest $\mathcal {C}^{fit}$ score is selected, the performances of MO will improve in these circumstances, but it will perform worse in other situations. This indicates an adaptive strategy for selecting splitting point from the *ε*-optimal set can further improve the performance.

**Fig. 7:**
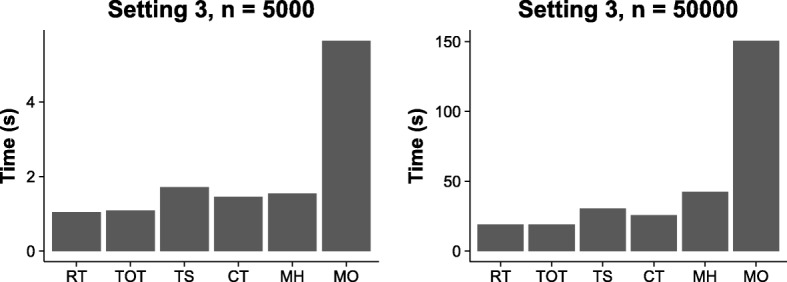
Running time comparison

The computational efficiency of MH is the same as existing methods. During the searching procedure, MH simply replaces the computation of fitness criterion by the heterogeneity criterion. For MO, the multi-objective search procedure introduces additional computation cost; however, because the Theorem guarantees that the upper bound of the cardinality of *S*^∗^ is a small constant, the running time of MO is within the same magnitude of other methods. Figure 7 shows the running time of all compared methods using Setting 3 with two sample sizes *n*=5000 and *n*=50000. The results here are the average execution time of 100 runs using a PC with a 3.4GHz single core CPU and 16GB of RAM. The time complexity of MH is similar to those of CT and TOT.

**Fig. 8:**
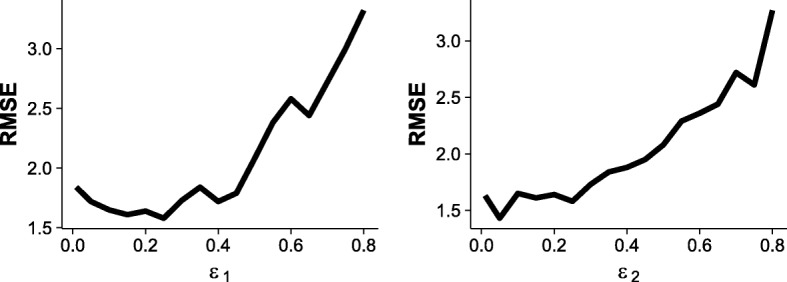
Influence of the parameters *ε*_1_ and *ε*_2_

Different choices of *ε*_1_ and *ε*_2_ values have influence on the performance of MO. It is worth noting that although the possible range of *ε* is from 0 to 1, small *ε* values should almost always be chosen since the effect of *ε* is proportional to the amount of slack in the *ε*-dominance relationship. Figure 8 illustrates how the parameter affects the performances in Setting 2 at sample size *n*=5000. In the left panel of Fig. 8, the value of *ε*_2_ is fixed at 0.05 and the value of *ε*_1_ varies; in the right panel the value of *ε*_1_ is fixed and the value of *ε*_2_ varies. The experimental results indicate that the algorithm generally achieves good performance when the *ε* ranges from 0.05 to 0.2.

### Heterogeneous treatment effects of radiotherapy in breast cancer patient

Understanding treatment effect heterogeneity has an important impact on the life quality of cancer patients. More than 50% of the breast cancer patients have received the radiotherapy treatment, equating to over half a million patients worldwide each year. Although radiotherapy is effective for many patients, not all of them benefit from the treatment [15].

We apply previously mentioned methods to study the treatment effect heterogeneity of radiotherapy on breast cancer patients. The data is obtained from the Cancer Genome Atlas (TCGA) [16]. The radiotherapy status is used the as the treatment indicator, the gene expression profiles are used as covariates, and the relapse-free survival status is used as the outcome.

Comparison of CATE estimation algorithms on real-world data is not straightforward because the ground truth treatment effects are not observable and the sample sizes are not large enough to divide the original data into training and testing sets.

An independent collection of 3951 breast cancer patients [17] is used for performance evaluation by examining how well the genes selected by each method can differentiate the survival probability between the radiotherapy treated and the untreated patients.

In Table 1 we compare the methods using the *p*-values calculated with log-rank test [18] and the combined *p*-values calculated with the Fisher’s method [19]. Smaller *p*-values indicates that the selected genes are more closely related to the survival probability of breast cancer patient. Considering the limited sample size, we restrict the maximum tree size to 4 terminal nodes for each method.

**Table 1:** *p*-values comparison on an independent breast cancer cohort

Method	1^*st*^ Split	2^*nd*^ Split	3^*rd*^ Split	Combined
RT	7 ×10^−09^	3 ×10^−4^	0.007	7 ×10^−12^
CT	1 ×10^−16^	5 ×10^−4^	0.017	1 ×10^−18^
TSTATS	1 ×10^−16^	5.4 ×10^−4^	0.017	1.1 ×10^−18^
TOT	6.7 ×10^−06^	2.5 ×10^−4^	0.017	9.1 ×10^−09^
MH	1 ×10^−16^	1.9 ×10^−7^	0.003	9.0 ×10^−23^
MO	1 ×10^−16^	1.4 ×10^−10^	0.001	3.5 ×10^−26^

The columns correspond to the *p*-values calculated using the gene selected at the first, second, the third splitting point and finally all the genes in the tree model

Overall all genes selected by the compared methods are related to the heterogeneity of radiotherapy treatment effects since all the *p*-values are smaller than the significance threshold (*p*=0.05) in every case. However, as shown in the table, every gene selected by MO achieves the smallest *p*-value in all compared methods. It is clear that the genes chosen by MO are clearly the most significantly related to the survival outcomes of breast cancer patients.

An interesting observation is that four of the six methods have chosen FOXF1 as the first gene to split, indicating that FOXF1 is closely related to breast cancer and the effectiveness of radiotherapy. In biology research, FOXF1 has been recently identified as important cancer-related gene [20]. Our findings could suggest a new direction for exploring its genetic function and contribution in cancer development.

Overall, the above results suggest that heterogeneous treatment effect estimation methods can be quite helpful in identifying the responsible genes for the differentiated response to a cancer treatment. The genes discovered by the proposed MO criterion has higher consistency in the independent test data than those discovered by other methods.

**Fig. 9:**
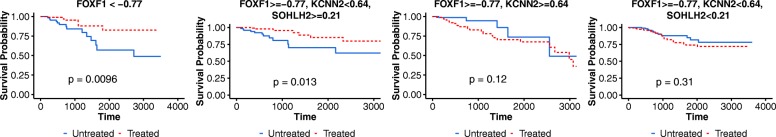
Heterogeneous treatment effects of radiotherapy on breast cancer patients

The treatment effect heterogeneities discovered by MO is illustrated in Fig. 9, where each panel shows the survival curves comparison between patients with radiotherapy treatment and those without the treatment for each of the terminal nodes. For those patients that are categorized into the first and the second subgroups, their estimated treatment effects of radiotherapy are 0.22 and 0.20, respectively. As evidenced by the *p*-values, the survival probability of the treated patients is significantly higher than the untreated ones. In other words, patients with low FOXF1 gene expression, and those with high FOXF1 and SOHLH2 expression but low KCNN2 expression, have benefited significantly from radiotherapy treatment. However, those patients in the third and the last subgroups do not benefit from radiotherapy. Interestingly, according to their negative estimated treatment effects, the prognosis of their disease are likely to worsen following the radiotherapy treatment.

## Related works

**RT** [5]. Standard regression tree can be modified to estimate heterogeneous treatment effects [7]. Specifically, the tree is constructed using the CART algorithm, and the treatment effect $\hat {\tau }_{i}(\mathbf {x})$ is estimated according to Eq. 2 using the samples within the same leaf.

**Transformed outcome tree** [6]. Transformed Outcome Tree (TOT) is based on the insight that existing regression tree methods can be used to estimate treatment effect by utilizing a transformed version of the outcome variable $Y_{i}^{TOT} = Y_{i} \cdot (W_{i} - \pi) / (\pi \cdot (1-\pi))$ as the regression target. Because $\mathbb {E}[Y_{i}^{TOT} |\mathbf {x}] = \tau (\mathbf {x})$, standard regression tree can be applied to the transformed outcome where the estimation of the sample average of $Y_{i}^{TOT}$ within each leaf can be interpreted as the estimation of the treatment effects.

**Causal tree** [7]. Causal Tree (CT) seeks the splitting point using the fitness criterion, but it does not consider the heterogeneity. In addition, they propose to divide the training samples into two disjoint parts to avoid bias in the treatment effect estimation, where the first part is used for selecting split and the second part is used to estimate the treatment effects in the model.

**Squared t-Statistic tree** [8]. squared T-Statistic tree (TS) seeks the split with the largest value for the square of the t-statistic for testing the null hypothesis that the average treatment effect is the same in the two potential leaves. The criterion is defined as:

$$\mathcal{C}^{TS} = n \cdot \frac{\left(\tau_{L}-\tau_{R}\right)^{2}}{\sigma_{L}^{2} / n_{L} + \sigma_{R}^{2} / n_{R}}, $$

where *σ*^2^ is the variance of treated and untreated samples within a node.

$\mathcal {C}^{hete}$ criterion is different from the TS criterion. Because the sample size grows smaller as the tree grows, the statistical test used in [8] suffers from loss of power. Unless the subgroup treatment effects are quite large, this method often fails to detect the effects in subgroups [21]. In the experiments it has been demonstrated that the performance of TS degenerates significantly as the number of variables increases, whereas the performances of MH remain unaffected.

## Conclusion

In this paper, we demonstrate that the heterogeneity of treatment effects should be explicitly considered in the splitting procedure.

We proposed two splitting criteria, MH and MO. MH explicitly considers the heterogeneity of treatment effects, and MO is a multi-objective criterion which balances heterogeneity and fitness at the same time.

Experiment results indicate that MH achieves better performances than existing methods when the differences between treatment effects in underlying subgroups are large, but is prone to error when the differences grow small. When fitness and heterogeneity are both taken consideration, the MO criterion performs consistently better than all studied methods.
